# Mechanical Alloying Behavior and Thermal Stability of CoCrCuFeMnNi_x_ High-Entropy Alloy Powders Prepared via MA

**DOI:** 10.3390/ma16083179

**Published:** 2023-04-18

**Authors:** Baofeng Zhang, Ruifeng Zhao, Bo Ren, Aiyun Jiang, Chong Chen, Jianxiu Liu, Yajun Zhou

**Affiliations:** 1 Faculty of Engineering, Huanghe Science and Technology College, Zhengzhou 450063, China; zhangbf1979@163.com (B.Z.);; 2 School of Mechanical Engineering, Henan University of Engineering, Zhengzhou 451191, China

**Keywords:** high-entropy alloy powder, mechanical alloying, phase transition, vacuum annealing, thermal stability

## Abstract

CoCrCuFeMnNi_x_ (x = 0, 0.5, 1.0, 1.5, 2.0 mol, named as Ni_0_, Ni_0.5_, Ni_1.0_, Ni_1.5_, and Ni_2.0_, respectively) high-entropy alloy powders (HEAPs) were prepared via mechanical alloying (MA), and XRD, SEM, EDS, and vacuum annealing were used to study the alloying behavior, phase transition, and thermal stability. The results indicated that the Ni_0_, Ni_0.5_, and Ni_1.0_ HEAPs were alloyed at the initial stage (5–15 h), the metastable BCC + FCC two-phase solid solution structure was formed, and the BCC phase disappeared gradually with the prolonging of ball milling time. Finally, a single FCC structure was formed. Both Ni_1.5_ and Ni_2.0_ alloys with high nickel content formed a single FCC structure during the whole mechanical alloying process. The five kinds of HEAPs showed equiaxed particles in dry milling, and the particle size increased with an increase in milling time. After wet milling, they changed into lamellar morphology with thickness less than 1 μm and maximum size less than 20 μm. The composition of each component was close to its nominal composition, and the alloying sequence during ball milling was Cu→Mn→Co→Ni→Fe→Cr. After vacuum annealing at 700~900 °C, the FCC phase in the HEAPs with low Ni content transformed into FCC2 secondary phase, FCC1 primary phase, and a minor σ phase. The thermal stability of HEAPs can be improved by increasing Ni content.

## 1. Introduction

A high-entropy alloy (HEA) is a new alloy system designed by using the strategy of equal or near-equal atomic ratio and high mixing entropy, and it is also one of the three breakthroughs in modern metal materials [1,2,3]. The four core effects of an HEA produced via the combination of a variety of main elements, namely, high mixing entropy, lattice distortion, slow diffusion and cocktail effect, have resulted in many microstructures and properties different from traditional alloys, such as simple solid solution structure, high strength, good low-temperature plasticity, good corrosion resistance and oxidation resistance, and good physical properties [4,5,6,7,8]. HEA systems containing Co, Cr, Cu, Fe, Mn, and Ni have been widely studied. Their preparation and processing technologies mainly include arc melting [1,2], induction melting [9], thermal mechanical processing [10,11], mechanical alloying [12,13,14], hot pressing sintering [15,16], plasma sintering [17], laser surface cladding [18], water atomization [12], etc. In the alloy systems studied, the CoCrFeMnNi alloy has a single FCC solid solution phase, good thermodynamic stability, and excellent ductility, which was reported by Cantor [1], Otto [19], and Gali [20]. In addition, the CoCrFeMnNi alloy shows an excellent combination of high strength and large elongation, as well as excellent fracture toughness at room temperature and low temperature [21,22,23,24,25,26]. The CoCrCuFeNi alloy prepared by high-pressure torsion (HPT) and annealing shows two FCC phases (Cu-rich phase and Cu-lean phase) and high aging hardness of 540 HV at 600 °C [27]. Hsu et al. [28] studied the corrosion behavior of the CoCrFeNiCu_x_ alloy in 3.5% NaCl solution and found that the CoCrCuFeNi alloy showed poor corrosion resistance due to interdendritic Cu segregation. The CoCrCuFeNi alloy exhibits excellent deformability due to phase separation into ductile FCC phases [29].

Casting is the main method used to produce the above HEAs. However, component segregation (such as Cu-rich phase) and volatilization of Mn are often observed during casting [30,31]. MA is considered as a solid-state processing method that can alloy elemental powders at room temperature. It has been widely used in the preparation of advanced materials. Uniform microstructure and ultra-fine/nanocrystalline structure can be easily obtained by controlling appropriate ball milling process parameters, which will certainly expand the application field of HEAs [32,33].

In our previous work, we prepared Co_x_CrCuFeMnNi and CoCr_x_CuFeMnNi HEAPs using the MA process, studied their alloying behavior and magnetic properties, and found that some HEAPs have excellent soft magnetic properties and thermal stability [14,34,35]. Ni is also one of the main factors affecting the alloying behavior and properties of HEAs. The increase in Ni content can inhibit the formation of the intermetallic compound phase in the HEAs and promote the formation of an FCC phase, which is conducive to improving the plasticity and corrosion resistance of the HEAs [36,37]. The effect of Ni on the phase structure of stable HEAPs has not been reported, especially at high temperatures. The high-temperature stability of HEAPs is very important for the microstructure and properties of sintered alloys. Therefore, in this study, a mechanical alloying method was used to prepare CoCrCuFeMnNi_x_ HEAPs, and their alloying behavior and thermal stability were emphatically studied in order to understand the effect of Ni on the microstructure of stable HEAPs, which will lay a preliminary foundation for the impact of powder superalloys on the microstructure and properties.

## 2. Materials and Methods

CoCrCuFeMnNi_x_ (x = 0, 0.5, 1.0, 1.5, 2.0 mol, named as Ni_0_, Ni_0.5_, Ni_1.0_, Ni_1.5_, and Ni_2.0_, respectively) HEAPs were prepared via MA. The purity of Co, Cr, Cu, Fe, Mn, and Ni metal powder higher than 99.9 wt.% was provided by Beijing Guanjinli New Materials Co., Ltd. (Beijing, China), with an average particle size of about 75 μm. The metal powders prepared according to the stoichiometric ratio (M_Ni_:M_Co_:M_Cr_:M_Cu_:M_Fe_:M_Mn_ = x:1:1:1:1:1, x = 0, 0.5, 1.0, 1.5, 2.0, mol, M stands for moles.) were put into 304L stainless-steel vacuum ball mill vials with a ball-to-material ratio of 10:1. The material of stainless-steel mill balls is also 304 L, and the mass ratio of large balls to small balls is 1:1, with diameters of 10 mm and 5 mm, respectively. After being purged with high-purity argon for 5 min, they were installed on a QM-WX4 planetary ball mill (Nanjing Nanda Instrument Co., Ltd., Nanjing, China) for mechanical alloying. The rotational speed of the ball mill was 300 rpm. The working time of the ball mill was 30 min, with an interval of 20 min. The HEAPs were first dry-milled for 45 h, and a small amount of powder was taken at 0 h, 5 h, 10 h, 15 h, 30 h, and 45 h to study the phase evolution of the powders. After the dry grinding, an appropriate amount (about 20 mL) of anhydrous ethanol was added and wet ground for 5 h; then, we took the powders and placed them in a vacuum-drying oven for drying treatment. The treatment temperature was 50 °C and the time was 48 h. The dried HEAPs were annealed under vacuum conditions of 6 × 10^−3^ Pa and different temperatures (700 °C, 800 °C, and 900 °C) for 2 h to analyze the thermal stability.

An X-ray diffractometer (XRD, Bruker D8 ADVANCE, Bruker, Germany) with Cu-Kα radiation was used to analyze the phase evolution of HEAPs. The wavelength was 1.54056 Å, the operating tube voltage and tube current were 40 kV and 40 mA, respectively, and the scanning angle was 20~90° (2*θ*). The scanning speed was 10°/min and the scanning step was 0.02°. The morphology and composition of HEAPs were observed and analyzed under a scanning electron microscope (SEM, Quanta 250, FEI, Czech) equipped with EDAX energy dispersion spectrometer (EDS). Particle sizes of the HEAPs were statistically investigated using Malvern laser particle size analyzer (Mastersizer 3000, Malvern, UK). Differential scanning calorimetry (DSC, STA 449 F3, Netzsch, Germany) was used for thermal analysis in high-purity argon atmosphere. The HEAPs were scanned in an alumina crucible with a temperature range of 25–1200 °C and a heating rate of 10 °C/min.

## 3. Results and Discussion

### 3.1. XRD Analysis of HEAPs

**Figure 1:**
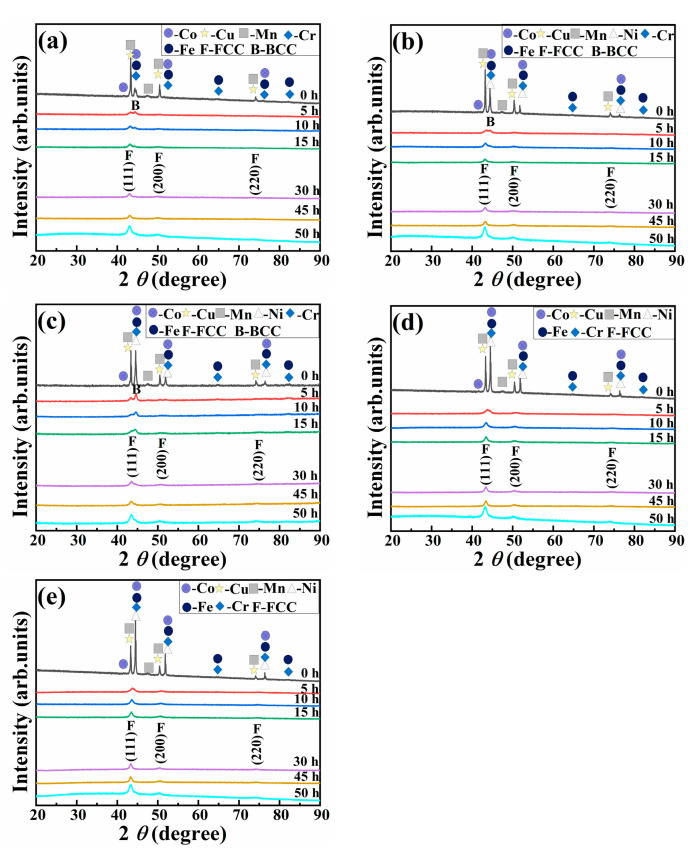
XRD patterns of CoCrCuFeMnNi_x_ HEAPs after different ball milling time: (**a**) Ni_0_, (**b**) Ni_0.5_, (**c**) Ni_1.0_, (**d**) Ni_1.5_, (**e**) Ni_2.0_.

Figure 1 shows the XRD analysis results of CoCrCuFeMnNi_x_ HEAPs after different ball milling times. From the XRD results of Ni_0_ HEAP (Figure 1a), it can be seen that the original metal powder presented two groups of strong diffraction peaks, respectively, corresponding to the diffraction peaks of Cr and Mn at 2*θ* = 44.60° and Cu, Fe and Co at 2*θ* = 43.34°. After 5 h ball milling, the diffraction peaks of Cu, Mn, and Co basically disappeared, indicating that these three metal elements and some Cr and Fe are a mutually solid solution, forming a solid solution with FCC structure as the main structure. Cu, Mn, and Co are the first elements to dissolve, which may be related to their relatively low melting points. After milling for 10 h, the diffraction peak intensity of each element further decreased. After 15 h ball milling, the diffraction peaks of Fe and Cr elements can still be detected in the XRD spectrum, but the diffraction peaks of two FCC phases can be clearly observed, which were located at 43.34° and 50.48°, respectively. After 30 h ball milling, all the diffraction peaks of pure elemental elements disappeared, and there were only three FCC phase diffraction peaks at different angles on the XRD spectrum. There were no significant differences between the diffraction peaks after 45 h ball milling and after 30 h. After 50 h ball milling (45 h dry milling + 5 h wet milling), the diffraction peaks in the FCC phase were obviously widened, indicating that when the alloying is completed, the grain of HEAPs will be refined by further increasing the ball milling time [38].

With an increase in milling time, the phase transition of Ni_0.5_ and Ni_1.0_ HEAPs was the same as that of Ni_0_ HEAP. After 5 h ball milling, the diffraction peaks of Cu, Mn, and Co elements disappeared, and the diffraction peaks of Cr, Fe, and Ni elements could still be observed. After 30 h and 45 h ball milling, a single FCC solid solution structure was formed. After 50 h ball milling, the diffraction peak width of the FCC phase increased significantly, and the HEAP grains were refined. The phase evolution of Ni_1.5_ and Ni_2.0_ HEAPs during 50 h ball milling was similar to that of Ni_0.5_ alloy powders, but the solid solution structure of Ni_1.5_ and Ni_2.0_ HEAPs after 5–30 h ball milling was a single FCC phase. The above results show that there are two main factors affecting the phase evolution of CoCrCuFeMnNi_x_ HEAPs during ball milling. One is the influence of alloying element content on its phase evolution, and the second is the influence of different milling time on the phase evolution. Alloying elements mainly affect the final phase structure of the HEAPs. For example, when Ni_0_ and Ni_0.5_ HEAPs were milled for 5–30 h, a solid solution structure was formed with the BCC phase as the main phase and FCC phase as the secondary phase, while Ni_1.5_ and Ni_2.0_ HEAPs were in a single FCC solid solution phase structure. Different ball milling time mainly affects the intensity and width of the diffraction peaks of CoCrCuFeMnNi_x_ HEAPs. At the beginning of ball milling, each metal element absorbed energy during the ball milling process and formed a solid solution structure with each other, leading to a weakening in the intensity of the diffraction peak. The extension of ball milling time and the addition of process control agent (wet milling for 5 h) led to a reduction in and broadening of the intensity of diffraction peaks.

According to the XRD results in Figure 1, the lattice parameters, grain size, lattice strain, and dislocation density of the FCC phase of Ni_0_ HEAP after different ball milling times can be calculated using Formulae (1)~(4). The lattice constant (*a*) is calculated via Equation (1):

(1) $$a=\frac{\lambda }{2sin\theta }\sqrt{h^{2}+k^{2}+l^{2}}$$

where *λ* is the X-ray wavelength (1.54056 Å), *θ* is the diffraction angle, and *h*, *k*, and *l* are Miller exponents. The grain size (*d*) and lattice strain (*ε*) of Ni_0_ HEAP are calculated via Equations (2) and (3).

(2) $$d=\frac{K\lambda }{\beta cos\theta }$$

(3) $$\varepsilon =\frac{\beta }{4tan\theta }$$

where *β* and *θ* are the measured half width of the spectral line and the diffraction angle of the corresponding peak position, respectively. *K* is taken as 0.89. The dislocation density (*ρ*) of the FCC phase in HEAPs can be calculated using Formula (4):

(4) $$\rho =\frac{2\sqrt{3}\varepsilon }{db}$$

(5) $$b=\frac{\sqrt{2}a}{2}$$

where *b* is the Burgers vector.

**Figure 2:**
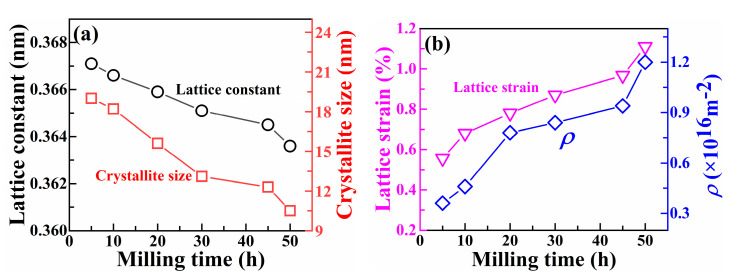
Relevant parameters of FCC phase in Ni_0_ HEAP after different ball milling time: (**a**) grain size and lattice constant; (**b**) lattice strain and dislocation density.

Figure 2 shows the relationship curve between milling time and lattice parameters, grain size, lattice strain, and dislocation density. It can be seen from Figure 2a that the lattice parameters and grain size of the FCC phase of Ni_0_ HEAP decreased with an increase in ball milling time. Figure 2b shows that the lattice strain and dislocation density increased with the increase in ball milling time. The grain size of the HEAP was between 9 and 20 nm, which indicates that the alloying elements form the FCC structure of nanocrystalline supersaturated solid solution under the action of mechanical alloying. Wang et al. [39] studied the alloying behavior of (CoCrFeNiMn) _90_M_10_ (M = Al, Hf) high-entropy alloy powder using the MA method and found that prolonged ball milling time resulted in a significant decrease in grain size and an increase in lattice strain, a phenomenon similar to the results of this study.

**Figure 3:**
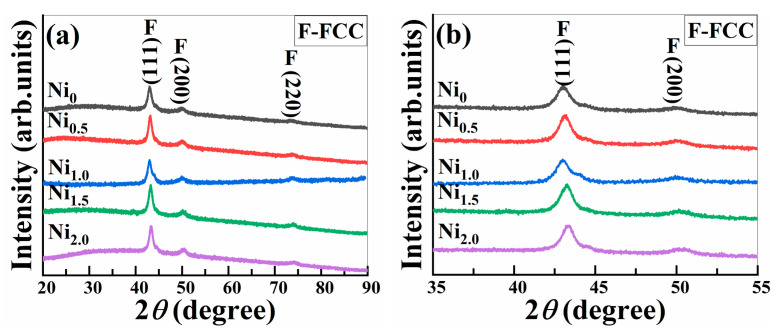
(**a**) XRD pattern of CoCrCuFeMnNi_x_ HEAPs after 50 h ball milling; (**b**) enlarged view of 35–55°.

Figure 3 shows the XRD pattern of CoCrCuFeMnNi_x_ HEAPs after 50 h ball milling. It can be seen that each HEAP has a single FCC solid solution phase structure. It can be observed from Figure 3b that with the increase in Ni content, the diffraction peak of the FCC phase gradually shifts to a large angle. The shift in the diffraction peak is generally affected by the grain size and lattice distortion. The addition of smaller Ni atoms will occupy the lattice points with larger atomic radius, making the lattice constant slightly smaller. According to the characteristics of HEAs, more constituent elements will increase the entropy value of HEAs, thus increasing the lattice distortion. As the atomic radius difference between each component atom in CoCrCuFeMnNi_x_ HEAs is small, its contribution to the shift in the diffraction peak is smaller than that of lattice distortion, so the diffraction peak shifts to a large angle with the increase in Ni content.

### 3.2. Microstructure of HEAPs

**Figure 4:**
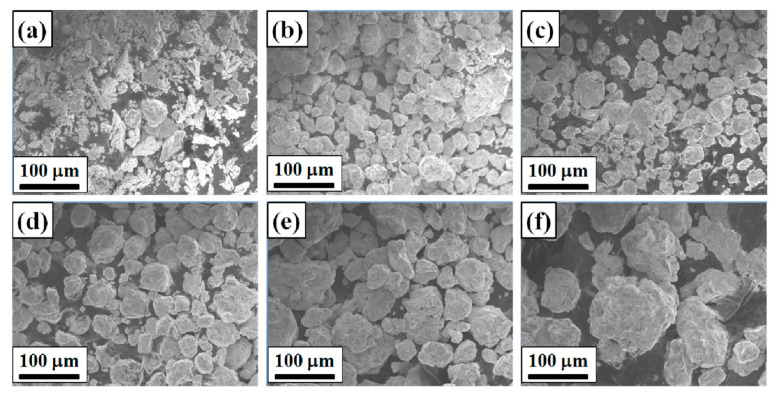
SEM photos of Ni_0_ HEAP after different ball milling times: (**a**) 0 h, (**b**) 5 h, (**c**) 10 h, (**d**) 15 h, (**e**) 30 h, (**f**) 45 h.

Figure 4 shows the SEM photos of the Ni_0_ HEAP after different ball milling times. It can be seen that the original powder after 0 h ball milling (Figure 4a) is in an irregular shape, and its particle size is less than 75 μm. Due to the intense impact and collision between the powder particles and the grinding balls, between the powder and the tank or between the powder and the powder under high-energy ball milling, the cold welding effect occurs, so the particle size of the HEAP increases significantly after 5 h (Figure 4b) and 10 h (Figure 4c) ball milling. With a further increase in ball milling time, the powder particles that are cold welded together will be broken into smaller particles, so the average particle size of the high-entropy alloy powder after 15 h ball milling (Figure 4d) is relatively small, and the shape of the powder tends to be equiaxed. In the process of ball milling, the powder particles repeatedly go through the cycle of extrusion deformation–welding–crushing, so that their grains are continuously refined, thus obtaining fresh crystal faces, increasing the contact area of the atomic reaction, shortening the diffusion distance between atoms, and promoting the formation of alloying. However, due to the agglomeration between the powders, the particle size of the powder milled for 30 h (Figure 4e) and 45 h (Figure 4f) is relatively large.

**Figure 5:**
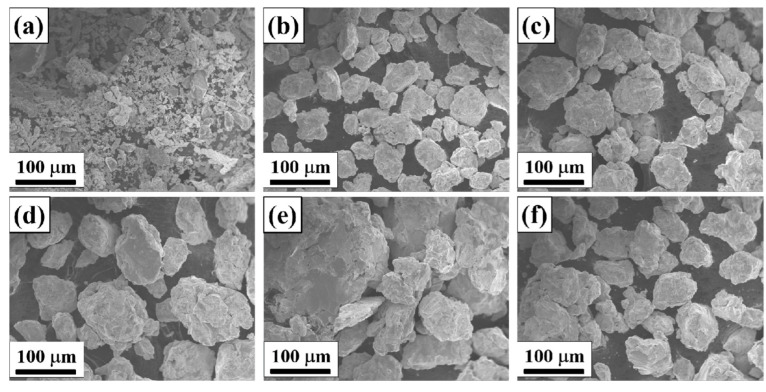
SEM photos of Ni_1.5_ HEAP after different ball milling times: (**a**) 0 h, (**b**) 5 h, (**c**) 10 h, (**d**) 15 h, (**e**) 30 h, (**f**) 45 h.

**Figure 6:**
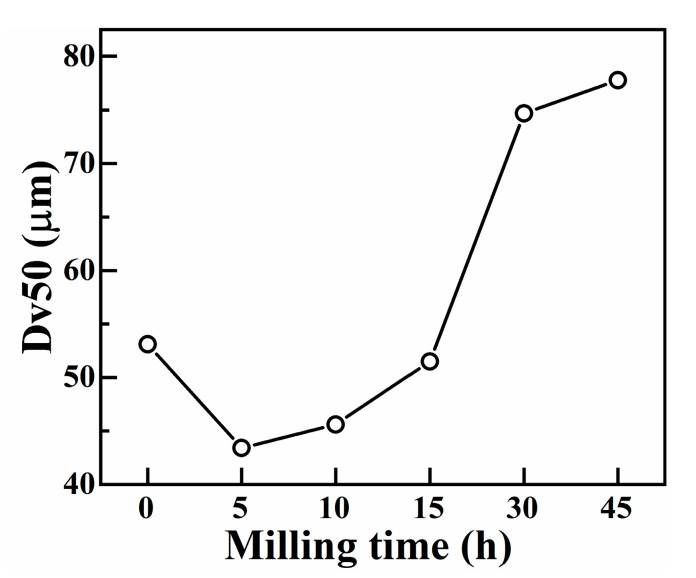
Dv50 of CoCrCuFeMnNi_1.5_ HEAP under different milling times.

Figure 5 shows the SEM photos of the Ni_1.5_ HEAP after different ball milling times. Figure 6 shows the median diameter (Dv50) of the Ni_1.5_ HEAP after different ball milling times. It can be seen from Figure 5a that the micromorphology of the Ni_1.5_ HEAP after 0 h ball milling is similar to that of Ni_0_ HEAP, which also presented an irregular shape. As can be seen from Figure 6, the particle size of Ni_1.5_ powder at 0 h was about 53 μm. After 5 h ball milling (Figure 5b), the micromorphology of the Ni_1.5_ HEAP was close to a nearly spherical particle shape, and the average particle size was reduced to 43 μm. After 10 h and 15 h ball milling (Figure 5c,d), the particle size increased to about 46 μm and 52 μm. After ball milling for 30 h (Figure 5e), the particle size of the powder particles further increased to 75 μm and showed agglomeration. After ball milling for 45 h (Figure 5f), the size of agglomerated particles further increased by about 78 μm. The micromorphology of Ni_0.5_, Ni_1.0_, and Ni_2.0_ HEAPs during mechanical alloying was similar to that of Ni_0_ and Ni_1.5_ alloy powders.

**Figure 7:**
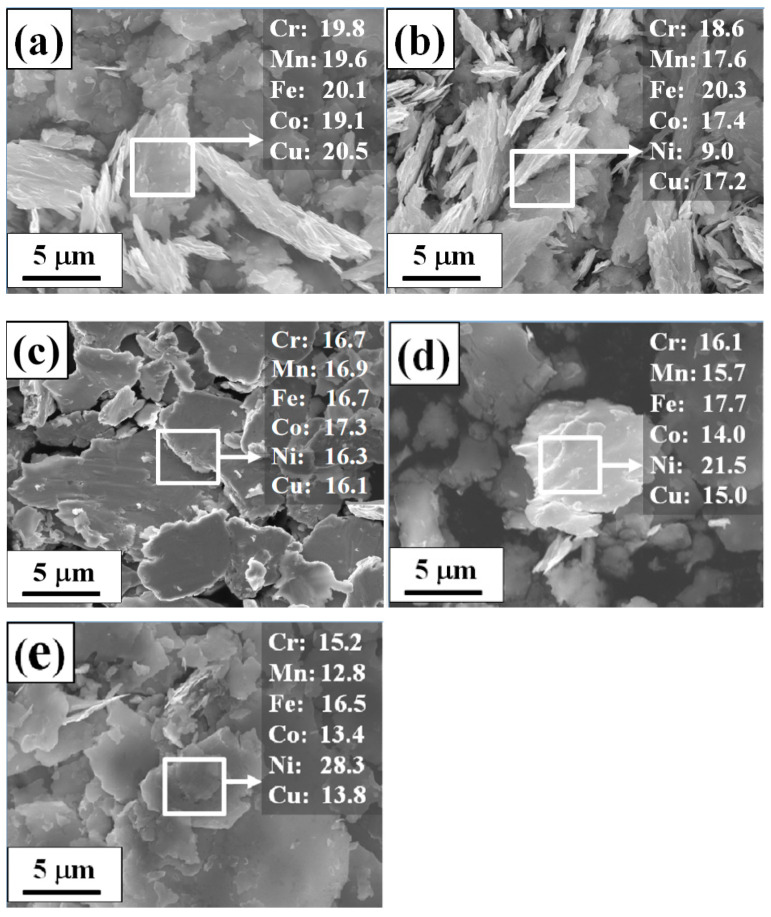
SEM photos of CoCrCuFeMnNi_x_ HEAPs after 50 h ball milling: (**a**) Ni_0_, (**b**) Ni_0.5_, (**c**) Ni_1.0_, (**d**) Ni_1.5_, (**e**) Ni_2.0_.

**Figure 8:**
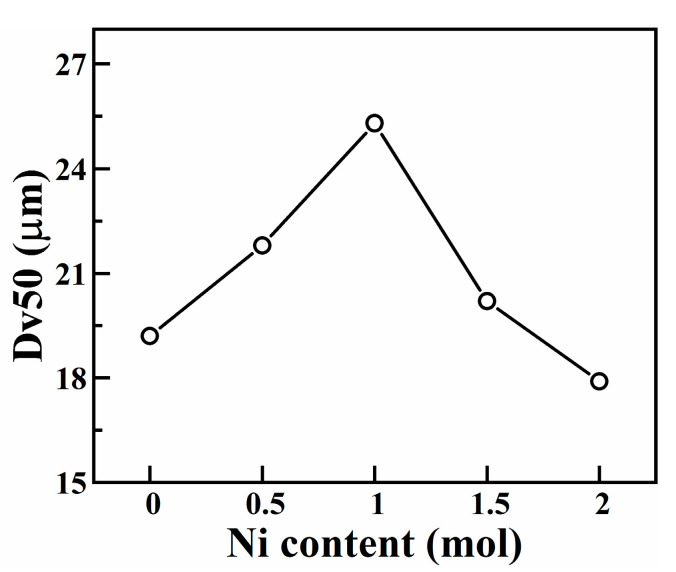
Dv50 of CoCrCuFeMnNi_x_ HEAPs after 50 h ball milling.

Figure 7 shows the SEM morphology and energy spectrum analysis results of CoCrCuFeMnNi_x_ HEAPs after 50 h ball milling. Figure 8 shows the Dv50 of CoCrCuFeMnNi_x_ HEAPs after 50 h ball milling. It can be seen that the micromorphology of the alloy powders after wet milling was irregular lamellar, and the thickness was less than 1 μm. As can be seen from Figure 8, with the increase in Ni content, the particle size of HEAPs first increased from 19 μm to 25 μm and then decreased to 18 μm, but the reason is still unclear. EDS analysis shows that the composition of each HEAP was close to its nominal composition. Therefore, CoCrCuFeMnNi_x_ HEAPs with fine grains and uniform chemical composition can be successfully prepared via MA. Mechanical alloying can effectively enhance the solid solubility behavior of alloying elements, thereby improving the solid solubility of constituent elements and forming a more stable phase structure [39,40].

### 3.3. Mechanical Alloying Behavior of HEAPs

During the whole process of mechanical alloying of CoCrCuFeMnNi_x_ HEAPs, except that Ni_1.5_ and Ni_2.0_ powders are always single FCC solid solution phase structure, the other alloys are all transited from the FCC + BCC solid solution phase to the single FCC phase. In the binary phase diagram, most of the components in equilibrium are finite solid solutions, so these two solid solutions are supersaturated solid solutions. The increase in solid solubility is closely related to milling time. With the extension of milling time, the severe deformation of the powder leads to a large number of structural defects and higher lattice strain, which can effectively improve the diffusion rate between atoms and the solubility of the solid solution. Equation (6) is the thermodynamic expression:

Δ*G_mix_* = Δ*H_mix_* − *T*Δ*S_mix_* (6)

where *T* is the absolute temperature, Δ*G_mix_* is the Gibbs free energy, and Δ*H_mix_* and Δ*S_mix_* are the mixing enthalpy and mixing entropy, respectively. Thermodynamic theory indicates that Δ*H_mix_* (**|**Δ*H_mix_***|**) represents the ordering and segregation trend of alloying elements, and Δ*S_mix_* represents the ability of random distribution of alloying elements in the lattice. Therefore, according to Formula (6), **|**Δ*H_mix_***|** and Δ*S_mix_* can be used to evaluate the resistance and driving force of solid solution formation. In the process of mechanical alloying, nanocrystals gradually form, resulting in a large number of grain boundaries, which can store a large number of Δ*H_mix_*, so the contribution of Δ*H_mix_* to Δ*G_mix_* can be ignored. The high positive value of *T*Δ*S_mix_* can significantly reduce Δ*G_mix_*, so the solid solution phase will be preferentially formed in the mechanical alloying process.

Table 1 lists the mixing enthalpy (Δ*H_mix_*), mixing entropy (Δ*S_mix_*), atomic size difference (*δ*), thermodynamic parameters (*Ω*), and valence electron concentration (*VEC*) of the CoCrCuFeMnNi_x_ HEA system calculated by Formula (7)~(11). Their calculation formulae are as follows:

(7) $$\Delta H_{mix}=\sum_{i=1,i\neq j}^{n}4\Delta H_{ij}^{mix}c_{i}c_{j}$$

(8) $$\Delta S_{mix}=-R\sum_{i=1}^{n}c_{i}lnc_{i}$$

(9) $$\delta =\sqrt{\sum_{i=1}^{n}c_{i}{\left(1-r_{i}/(\sum_{i=1}^{n}c_{i}r_{i})\right)}^{2}}$$

(10) $$\Omega =\frac{\sum_{i=1}^{n}c_{i}{\left(T_{m}\right)}_{i}∆S_{mix}}{\left|∆H_{mix}\right|}$$

(11) $$VEC=\sum_{i=1}^{n}c_{i}({VEC)}_{i}$$

where *n* is the elemental number of alloys, *c_i_* and *c_j_* are the atomic percentages of the *i*th and *j*th elements, $\Delta H_{ij}^{mix}$ is the mixing enthalpy of binary liquid alloy, and their values can be obtained in reference [41]. R is the gas constant, *r_i_* is the atomic radius of the *ith* element, which can be obtained from reference [42]. (*T_m_*)*_i_* and (*VEC*)*_i_* are the melting point and valence electron concentration (VEC) of the *i*th element, respectively.

According to the solid solution formation criteria shown in Table 2, the mixing enthalpy Δ*H_mix_*, mixing entropy Δ*S_mix_*, *δ*, and *Ω* of CoCrCuFeMnNi_x_ HEA are all within the range of solid solution formation criteria, so the alloy system tends to form a solid solution phase. In addition, the *VEC* values in the alloy system are greater than 8, and its phase tends to form a single FCC structure, which is consistent with the XRD results in Figure 3. It can be seen that the valence electron concentration criterion is suitable for predicting the formation of solid solution phase in the CoCrCuFeMnNi_x_ HEA system.

**Figure 9:**
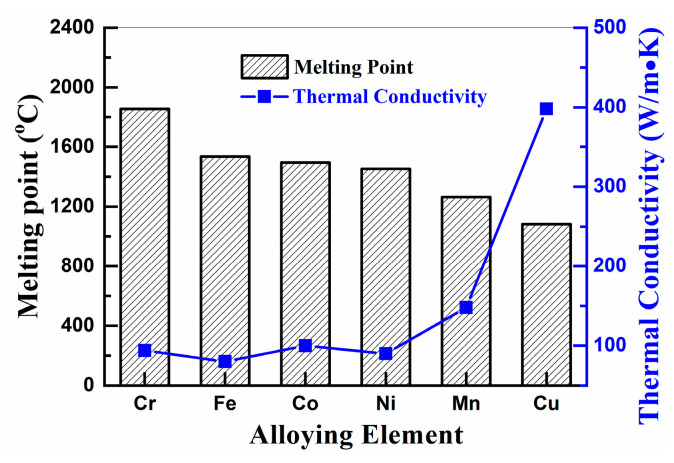
Melting point and thermal conductivity of elements in CoCrCuFeMnNi HEA system [14].

The phase formation mechanism of HEAPs is mainly related to the alloying behavior of components during mechanical alloying. Due to the small difference in physical properties and electronegativity of elements in the CoCrCuFeMnNi_x_ HEA system, the alloying process is mainly diffusion-controlled. The diffusion rates of alloying elements are different due to their physical properties. Chen et al. [48] showed that elements with a low melting point or brittle crystal structure may be preferentially dissolved during ball milling. Figure 9 shows the melting point and thermal conductivity of each element in the CoCrCuFeMnNi HEA system [14]. The melting points of Co and Ni are very similar, but Ni with an FCC phase has higher plasticity than Co with an HCP phase, so the alloying of Co occurs before Ni. Therefore, the alloying sequence of CoCrCuFeMnNi_x_ HEAPs during ball milling is Cu→Mn→Co→Ni→Fe→Cr.

Generally, the solid solubility of an alloy is mainly affected by the following three factors: (1) crystal structure, (2) the atomic size of the constituent elements (i.e., the atomic size difference), (3) chemical compatibility between constituent elements (i.e., electronegativity difference or Δ*H_mix_*). In the process of mechanical alloying, the dissolution of components may be mainly affected by the crystal structure and atomic size difference, while the chemical compatibility between components has little influence on it, because a large number of mixing enthalpies are limited in the grain boundary of nanocrystals. Therefore, during mechanical alloying of CoCrCuFeMnNi_x_ HEAPs, Cu and Mn elements are the first to finish alloying. In addition, some studies have shown that in the process of mechanical alloying, elements with similar crystal structure and atomic size often dissolve each other to form solid solutions [49]. Therefore, when Ni is contained in the high-entropy alloy, the Cu and Mn elements that first completed alloying may dissolve into the Ni element, forming an FCC solid solution structure with the same crystal structure as the Ni element. In addition, Praveen et al. [50] also showed that Ni can be used as a solvent for the FCC solid solution phase, because the melting point of Ni is higher than that of Cu and Mn, and the diffusion rate is lower than that of Cu and Mn. Similarly, most Fe atoms can be dissolved into Cr as a solvent to form a BCC solid solution phase structure. Due to the HCP structure of Co (closely packed hexagonal crystal structure), its alloying behavior may be affected by the atomic size difference and chemical compatibility, which are different from the other five constituent elements. If the influence of atomic size difference is taken into account, Co atoms tend to dissolve into Ni to form the FCC solid solution phase, because the atomic size difference between Co and Ni is smaller than that between Co and Cr. If chemical compatibility between component elements is considered, Co atom tends to dissolve into Cr, because Co-Cr has the most negative mixing enthalpy (Table 3), and the two have a strong binding force.

However, the research on Co_x_CrCuFeMnNi HEAPs shows that an increase in Co is beneficial to the formation of the FCC solid solution phase [14]. The research of Fu et al. [51] also shows that the elimination of Co will reduce the FCC solid solution phase in Al_0.6_NiFeCrCo HEA. It can be inferred that in the CoCrCuFeMnNi_x_ HEA system, the number of Co atoms dissolved in Ni is greater than that in Cr. In addition, it can be seen from the XRD spectrum shown in Figure 1 that after 5 h of ball milling, except for the weakening of the corresponding diffraction peaks of Cr and Fe, the diffraction peaks of other alloying elements basically disappeared, forming a solid solution structure dominated by BCC. After prolonging the milling time (30 h), due to the high-entropy effect and the solid solubility expansion caused by mechanical alloying, this unstable BCC solid solution phase is decomposed, and then the FCC structure dominated by Cu is formed. At this time, the competition mechanism in the CoCrCuFeMnNi_x_ HEA system will play a leading role; that is, the amount of FCC-stable elements and BCC-stable elements will determine the final stable phase of the alloy system: when the current content exceeds the latter and the difference is not large, the FCC + BCC mixed phase will be formed; when the current content exceeds the latter and the difference is large, a single FCC phase will be formed. Therefore, after 50 h ball milling, CoCrCuFeMnNi_x_ HEAPs all have a single FCC solid solution structure. However, Ni_1.5_ and Ni_2.0_ HEAPs are always single FCC phase during ball milling, and there is no decomposition of BCC phase, which is mainly affected by the competition mechanism. During the ball milling process, Cu, Mn, Co, and Ni are the first to complete the alloying, generating a large number of FCC solid solution phases with structural defects and high lattice strain. When Fe and Cr elements are decomposed, the FCC solid solution phase with a large number of defects will preferentially dissolve Fe and Cr atoms, so Ni_1.5_ and Ni_2.0_ HEAPs are always single FCC solid solution structures. In conclusion, the main composition of the FCC solid solution phase in CoCrCuFeMnNi_x_ HEAPs is Ni (Cu, Mn, Co), and the main composition of the BCC solid solution phase is Cr (Fe, Co).

### 3.4. Thermal Stability of HEAPs

**Figure 10:**
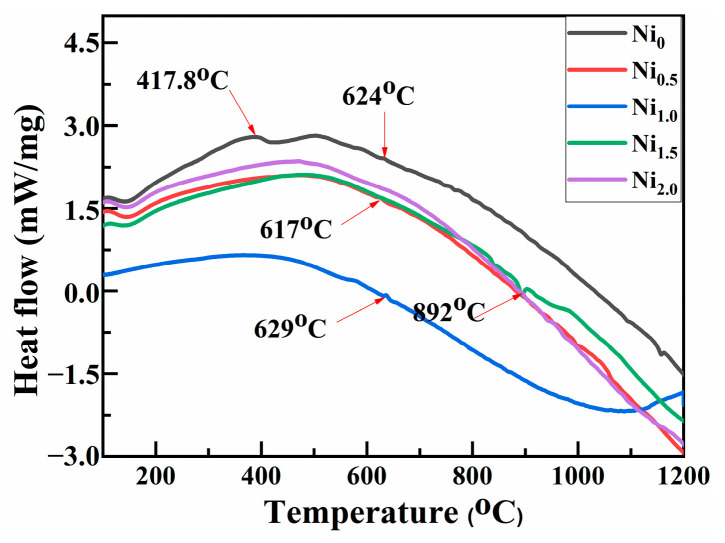
DSC Curves of CoCrCuFeMnNi_x_ HEAPs after 50 h ball milling.

Figure 10 shows the DSC curve of CoCrCuFeMnNi_x_ HEAPs after 50 h ball milling. It can be seen that the trend of DSC curves of the five HEAPs is basically consistent, and there are long exothermic curves in a range from room temperature to 400 °C, which is related to the increase in the lattice strain of alloy powders and the release of internal stress such as crystal structure transformation. The smaller exothermic peaks of Ni_0_, Ni_0.5_, Ni_1.0_, and Ni_1.5_ HEAPs can be observed at 624 °C, 617 °C, 629 °C, and 892 °C, respectively. However, no obvious exothermic peak was found in the Ni_2.0_ HEAP. The appearance of a small exothermic peak in DSC curves of HEAPs may be related to the release of internal stress at high temperature and the phase transformation of the supersaturated solid solution.

**Figure 11:**
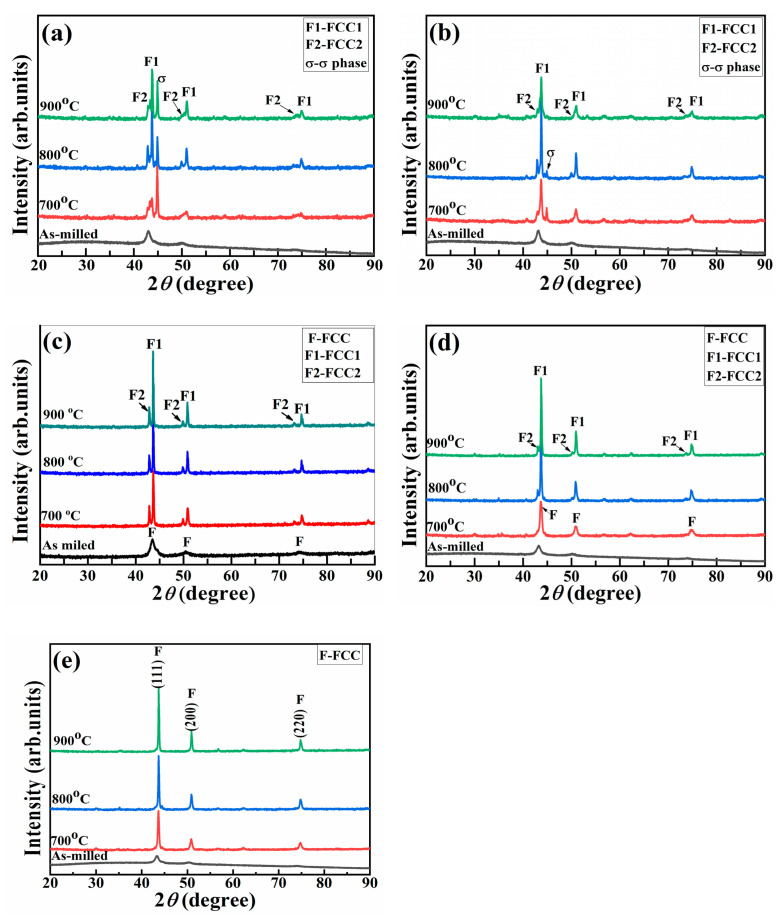
XRD results of CoCrCuFeMnNi_x_ HEAPs after vacuum annealing at different temperatures: (**a**) Ni_0_, (**b**) Ni_0.5_, (**c**) Ni_1.0_, (**d**) Ni_1.5_, (**e**) Ni_2.0_.

According to the DSC curve results, the XRD results of the CoCrCuFeMnNi_x_ HEAPs milled for 50 h after 2 h vacuum annealing at 700 °C, 800 °C, and 900 °C are shown in Figure 11. It can be seen from Figure 11a that the Ni_0_ HEAP had a single FCC solid solution phase structure in the ball milling state. After annealing at 700 °C, the FCC phase decomposed into a Cu-rich FCC2 phase, a heavy Cr-rich σ phase, and slightly Cr-rich FCC1 phase. After annealing at 800 °C, the relative contents of the three changed, and after annealing at 900 °C, there was no significant change compared with that of 800 °C. The phase transformation of the Ni_0.5_ HEAP after annealing at 700~900 °C is roughly the same as that of the Ni_0_ HEAP (Figure 11b). After annealing at three temperatures, the FCC phase of the Ni_1.0_ HEAP was decomposed into FCC1 and FCC2 phases and no obvious σ phase (Figure 11c). Ni_1.5_ HEAP had no obvious phase transformation after vacuum annealing at 700 °C, but the FCC phase was also decomposed into FCC1 and FCC2 phases after annealing at 800 °C and 900 °C (Figure 11d). Ni_2.0_ HEAP had no obvious phase transformation after annealing at 700~900 °C (Figure 11e). This shows that the FCC phase in CoCrCuFeMnNi_x_ HEAPs will be transformed into FCC1 primary phase, FCC2 secondary phase, and a minor *σ* phase after annealing at a certain temperature. It can be seen that the FCC phase formed by mechanical alloying is a metastable supersaturated solid solution phase, which will undergo phase transformation during high-temperature annealing. In the Co_x_CrCuFeMnNi HEAP system, an increase in Co content led to an increase in the metastable FCC solid solution content of the alloy. After high-temperature vacuum annealing, the FCC phase decomposed into two solid solution phases, FCC1 and FCC2, with a more stable structure [34]. However, Ni seems to have higher FCC stability because Ni_2.0_ HEAP still retains its original phase structure without phase separation after vacuum annealing at 700–900 °C, thereby exhibiting excellent thermal stability. Therefore, the increase in Ni content can effectively improve the stability of the FCC phase in HEAPs and avoid phase transformation. 

## 4. Conclusions

CoCrCuFeMnNi_x_ HEAPs were prepared via MA to generate materials with nanocrystals with metastable structures. The effects of milling time and vacuum annealing temperature on the mechanical alloying behavior, phase transformation, and thermal stability were studied. When the milling time was less than 15 h, the three HEAPs Ni_0_, Ni_0.5_, and Ni_1.0_ consisted of metastable BCC + FCC phases. After ball milling for more than 30 h, the three HEAPs all formed FCC phases with a more stable structure. Ni_1.5_ and Ni_2.0_ HEAPs were composed of a single FCC phase with a more stable structure during the entire ball milling process. Five kinds of HEAPs formed nearly spherical particles after dry grinding and formed a lamellar morphology after wet grinding. The particle size was finer, and the element content was close to the nominal composition. HEAPs with low Ni content transformed from the FCC phase to FCC1 + FCC2 + *δ* (Ni_0_ and Ni_0.5_) phases or FCC1 + FCC2 phases (Ni_1.0_) after vacuum annealing at 700–900 °C, while Ni_2.0_ powder remained a single FCC phase after high-temperature annealing, exhibiting excellent thermal stability, indicating that Ni content has an important stabilizing effect on the structure of the FCC phase in HEAs.

## Figures and Tables

**Table 1:** Thermodynamic parameters of CoCrCuFeMnNi_x_ HEA system.

Alloy	Δ*H_mix_*	Δ*S_mix_*	*Ω*	*δ*	*VEC*
Ni_0_	4.16	13.38	55.33	3.15	8.20
Ni_0.5_	2.58	14.70	98.05	3.07	8.36
Ni_1.0_	1.44	14.90	17.81	3.01	8.50
Ni_1.5_	0.615	14.78	41.38	2.92	8.61
Ni_2.0_	0	14.53	-	2.85	8.71

**Table 2:** Formation criteria of solid solution of HEAs.

Proponent	Empirical Rules
Hume-Rothery [43]	*f* ≥ 5, −40 < Δ*H_mix_* < 10 KJmol^−1^, d < 12%
Zhang [44]	−20 < Δ*H_mix_* < 5 KJmol^−1^, 12 < Δ*S_mix_* < 17.5 JK^−1^mol^−1^, *δ* < 6.4%
Yang [45]	*Ω* ≥ 1.1, *δ* ≤ 6.6%
Wang [46]	*γ* < 1.175
Guo [47]	FCC (*VEC* < 6.87), FCC + BCC (6.87 ≤ *VEC* < 8), BCC (*VEC* ≥ 8)

**Table 3:** Mixing enthalpy of binary liquid alloy of the CoCrCuFeMnNi HEA system (KJ/mol) [42].

Element	Co	Cr	Cu	Fe	Mn	Ni
Co	0	−4	6	−1	−5	0
Cr	-	0	12	−1	2	−7
Cu	-	-	0	13	4	4
Fe	-	-	-	0	0	−2
Mn	-	-	-	-	0	−8
Ni	-	-	-	-	-	0

## Data Availability

All the raw/processed data required to reproduce these findings are available and can be requested from the authors.
